# A supervised machine learning approach to predicting plasma biomarkers of Alzheimer's disease among diverse Hispanic/Latino adults: Findings from the SOL‐INCA study

**DOI:** 10.1002/alz70860_105290

**Published:** 2025-12-23

**Authors:** Sayaka Kuwayama, Wassim Tarraf, Kevin A Gonzalez, Freddie Márquez, Natasha Z. Anita, Melissa Lamar, Humberto Parada, Ariana M Stickel, Carmen R Isasi, Paola Filigrana, Martha L Daviglus, Linda C Gallo, Hector M González

**Affiliations:** ^1^ University of California, San Diego, La Jolla, CA, USA; ^2^ Wayne State University, Detroit, MI, USA; ^3^ Rush Alzheimer's Disease Center, Rush University Medical Center, Chicago, IL, USA; ^4^ San Diego State University, San Diego, CA, USA; ^5^ Albert Einstein College of Medicine, Bronx, NY, USA; ^6^ University of Illinois at Chicago, Chicago, IL, USA

## Abstract

**Background:**

Plasma phosphorylated tau‐181 (*p*‐tau‐181) has emerged as a reliable blood‐based biomarker (BBM) for Alzheimer's disease (AD), reflecting tau pathology. Elevated *p*‐tau‐181 levels have been associated with amyloid beta deposition, AD progression and cognitive decline, and there is increasing support for use of *p*‐tau‐181 levels in disease diagnosis and monitoring. Understanding the predictors of *p*‐tau‐181 variability is necessary to enhance clinical utility, identify disease mechanisms, and refine targeted interventions.

US‐based Hispanic/Latino adults are disproportionately affected by AD and related dementias. Identifying predictors of the BBMs of AD neuropathologic change in this population may help identify evidence‐based targeted intervention points to modify the course of AD‐related pathologic processes. We implemented machine learning (ML) prediction models (Random Forest) to identify predictors of high *p*‐tau‐181 levels in a deeply characterized population of middle‐aged and older US‐based Hispanic/Latino adults.

**Method:**

We used data (*N* = 5,175, mean baseline age=56.4 years) from the Hispanic Community Health Study/Study of Latinos (2008‐2011), a prospective cohort study of US‐based Hispanics/Latinos, and its ancillary study, the Study of Latinos‐Investigation of Neurocognitive Aging (2015‐2018).

We categorized *p*‐tau‐181 as a binary measure (>90th versus ≤90th percentile) in the training dataset. We specified our ML models using 33 potential baseline predictors of *p*‐tau‐181 assessing life‐course risk factors reflective of the 2024 Lancet report on dementia prevention and used permutation techniques to assess factor importance.

**Result:**

The discriminative performance of the model in distinguishing between high and low *p*‐tau‐181 was moderate (area under the receiver operating characteristic curve=0.73) on the testing dataset. Among the identified predictors of high *p*‐tau‐181, the top predictors included diabetes, employment status, and the Short Acculturation Scale for Hispanics‐language subscale.

**Conclusion:**

Predictors of *p*‐tau‐181 in middle‐aged and older Hispanic/Latino adults include multiple life‐course factors. While these findings highlight the complexity of targeting and modifying disease risk in this population, the findings on the top predictors offer specific areas of focus including cardiometabolic risk and social determinants. Follow‐up work will replicate and validate these models focusing on distal and proximal measurements of *p*‐tau and changes in levels and will expand our findings to other BBMs of AD neuropathologic change.

